# The Role of Diet and Dietary Patterns in Parkinson’s Disease

**DOI:** 10.3390/nu14214472

**Published:** 2022-10-25

**Authors:** Emily Knight, Thangiah Geetha, Donna Burnett, Jeganathan Ramesh Babu

**Affiliations:** 1 Department of Nutritional Sciences, Auburn University, Auburn, AL 36849, USA; 2 Boshell Metabolic Diseases and Diabetes Program, Auburn University, Auburn, AL 36849, USA

**Keywords:** Parkinson’s Disease, diet, dietary patterns, ketogenic diet, Mediterranean diet, MIND diet, protein-restricted diet

## Abstract

Parkinson’s Disease (PD) is a neurodegenerative disorder associated with diminished nutrition status and decreased quality of life. While the prevalence of PD is expected to increase, no preventative or curative therapy for PD exists at this time. Although nutrition and diet represent modifiable risk factors for reducing chronic disease risk, research on the impact of single nutrients on PD has yielded mixed results. As a result, this single-nutrient approach may be the driving force behind the inconsistency, and a holistic dietary approach may overcome this inconsistency by accounting for the interactions between nutrients. The following review aims to examine the impact of a generally healthy dietary pattern, the protein-restricted diet (PRD), the ketogenic diet (KD), the Mediterranean diet (MD), and the Mediterranean-DASH Intervention for Neurodegenerative Delay (MIND) diet on PD risk, progression, and severity. While most of the included studies support the role of diet and dietary patterns in reducing the risk of PD or alleviating PD severity, the inconsistent results and need for further evidence necessitate more research being conducted before making dietary recommendations. Research on the potential beneficial effects of dietary patterns on PD should also investigate potential risks.

## 1. Introduction

With 680,000 men and women ages 45 and older having Parkinson’s Disease (PD) in the United States (US) in 2010, PD is the second most prevalent neurodegenerative disorder following Alzheimer’s Disease [1,2]. While the prevalence of PD correlates with geographical region and biological sex, the most significant risk factor is increasing age [1,2,3]. As the number of older adults increases in the US, PD cases will likely exceed 1.2 million by 2030 [1]. First identified by James Parkinson [4] in 1817, PD is a disorder characterized by its cardinal motor symptoms of bradykinesia, postural instability, rigidity, and tremors, along with the degeneration of dopaminergic neurons [5,6,7,8]. The histopathological hallmarks of PD—Lewy bodies and Lewy neurites—result from the intracellular aggregation of α-synuclein [9,10,11]. In addition, PD includes non-motor symptoms, such as cognitive decline, constipation, depression, hyposmia, and urinary dysfunction [12,13,14].

Since several years may pass before neurodegeneration manifests the motor and non-motor symptoms of PD, the International Parkinson, and Movement Disorder Society (MDS) broke the definition of PD down into three categories: preclinical PD, prodromal PD, and clinical PD [15,16,17]. These three categories differentiate individuals with PD-related neurodegeneration based on the presentation and the type of symptoms. For instance, prodromal PD denotes individuals with neurodegeneration between the presymptomatic stage of preclinical PD and the diagnostic stage of clinical PD [16,17,18,19,20]. As a result, the three categories distinguish between PD cases based on progression, but other tools differentiate PD cases based on severity.

Scientific research has primarily utilized two different tools for measuring PD severity. The first tool was developed by Hoehn & Yahr [21] in 1967. The scale comprises five stages for categorizing PD patients based on the degree of affected extremities and the severity of an individual’s disability. The second tool, the Unified Parkinson’s Disease Rating Scale (UPDRS), was revamped by the MDS in 2008 to create the MDS-UPDRS [22]. The MDS-UPDRS has four parts: Non-Motor Experiences of Daily Living (Part I), Motor Experiences of Daily Living (Part II), Motor Examination (Part III), and Motor Complications (Part IV) [22,23]. As a result, the MDS-UPDRS allows for a more thorough examination of PD severity than the UPDRS, based on the type of symptoms and the impact on activities of daily living.

Since PD progression is linked to increased disability and caregiver burden, the severity of PD has a notable impact on an afflicted individual’s quality of life [24,25,26]. Moreover, the COVID-19 pandemic may have exacerbated the burden of PD. Recent research indicates PD patients, regardless of COVID-19 infection history, experienced disrupted healthcare provision, worsened symptoms, and decreased quality of life [27,28,29]. As a result, reducing PD severity is becoming increasingly essential to improving the quality of life of individuals with PD.

In addition to disease severity, the nutritional status of an individual with PD is directly related to their quality of life [30,31,32]. A key indicator of nutrition status, malnutrition looks past an individual’s body mass index (BMI) and focuses on body composition, energy intake, and weight changes [33]. Furthermore, malnutrition is a concern for older adults due to its association with increased healthcare costs, length of stay, mortality, and readmission among hospitalized patients [34,35,36]. Notably, between 3 to 60% of individuals with PD are at malnutrition risk [37]. While malnutrition is a critical measure of nutrition status, an individual with PD’s motor and non-motor symptoms also influences nutrition status [38]. For instance, a three-year cohort study found a direct relationship between constipation and body fat loss [39]. Additionally, researchers observed a direct relationship between an individual’s risk of malnutrition and motor symptom severity [40,41]. Other nutrition-related symptoms may include dementia, depression, dyskinesia, dysphagia, gastroparesis, hyposmia, tremors, rigidity, and small bowel dysfunction [38,42,43,44,45]. As a result, both an individual’s motor and non-motor symptoms may impact their nutrition status and quality of life.

As PD’s exact etiology and pathophysiology remain unclear, researchers experience difficulty in developing effective preventative and curative strategies. At this time, no preventative or curative treatment for PD exists, but several pharmacological drugs may alleviate PD symptoms. Currently, levodopa is the most common pharmacological intervention for improving the motor symptoms of PD [46]. One approach to studying the development of PD is to explore factors associated with increased PD risk. For instance, several factors contribute to the risk of developing PD, including genetics, biological sex, environmental exposures, and lifestyle [47,48,49,50]. As a result, modifiable risk factors serve as potential targets for attenuating an individual’s risk.

Since nutrition and diet represent modifiable risk factors for other chronic diseases, they are potential areas for reducing PD risk and slowing disease progression. A meta-analysis observed that caffeine consumption typically from coffee is inversely related to PD risk and progression [51]. Nevertheless, several studies examining dietary antioxidants—polyphenols, carotenoids, vitamin A, vitamin C, and vitamin E—have yielded inconsistent results [52,53,54,55,56,57]. Likewise, studies on the role of dietary fat in PD yielded mixed results [58]. Thus, several researchers have proposed that the single-nutrient approach used to examine disease risk may be the driving force behind the inconsistency, arguing that a holistic approach could account for the synergistic effect [59]. Therefore, this review examined the impact of diet and dietary patterns on PD.

## 2. General Dietary Patterns

Dietary pattern is a broad term used to describe an individual or population’s intake of dietary components over a certain period [60,61]. Over the years, research has supported the role of a healthy dietary pattern in preventing or alleviating depression, dementia, and neurodegeneration [62,63]. Articles were identified using PubMed and Google Scholar, along with screening the reference sections of included articles. The studies included in this review were published in or after 2011 to focus on recent research on the effect of dietary patterns on PD, although older studies were identified in the published literature.

### 2.1. Defining a Healthy Dietary Pattern

A ‘healthy dietary pattern’ represents any dietary pattern scientifically linked to reduced mortality and improved health outcomes. As a result, great variety exists in defining a healthy dietary pattern. A broad range of scoring systems further complicates the definition of a healthy dietary pattern. For example, the Alternative Healthy Eating Index (AHEI) is a scoring system that assesses diet quality and adherence to the Dietary Guidelines for Americans [64,65]. Another scoring system, the Dietary Inflammatory Index (DII), measures an individual’s consumption of foods associated with inflammation [66]. Like other dietary indexes, the DII is associated with positive health outcomes, including a reduced risk of cardiovascular disease [66]. Consequently, a healthy dietary pattern links an eating style to a favorable health outcome.

While healthy dietary patterns generally focus on improving an individual’s overall health, several dietary patterns are geared toward specific diseases, food groups, or the timing of meals. First, dietary patterns may target the risk reduction of specific diseases and conditions. For instance, the Dietary Approaches to Stop Hypertension (DASH) diet is a dietary pattern characterized by the consumption of fruit, vegetables, and low-fat dairy products and reduced intake of sodium, saturated fat, and cholesterol [67,68]. As its name implies, the DASH diet represents a dietary pattern designed to assist individuals in managing their blood pressure [67,68]. Second, dietary patterns may reflect the composition of a meal. For example, vegetarian and vegan dietary patterns reflect the consumption of plant-based foods and beverages instead of animal products [69,70]. Third, dietary patterns may reflect the timing of meals. Time-restricted diets, like intermittent fasting, restrict eating to specific time intervals during a day or week [71,72].

### 2.2. The Impact of General Dietary Patterns on PD

Previous research examined the relationship between PD and an individual’s dietary intake of specific food groups. A cross-sectional study by Mischley et al. [73] evaluated the dietary patterns of 1053 US men and women with idiopathic PD. Mischley et al. [73] found a significant relationship between reduced PD severity and increased consumption of coconut oil, fish, fresh fruit, fresh vegetables, nuts, olive oil, spices, and wine. Likewise, PD severity decreased with the diminished consumption of beef, canned fruit, canned vegetables, cheese, yogurt, ice cream, fried food, and diet soda [73]. As a result, Mischley et al. [73] concluded that adherence to a plant-based diet with fish might slow the progression of PD. While other studies support the role of increased vegetable consumption in reducing PD risk, some studies have found that the type of dairy product consumed affects PD risk [74,75,76]. Although Hughes et al. [75] observed increased dairy and milk intake to be associated with an increased risk for PD, the authors noted that the type of dairy product could impact PD risk. PD risk increased with an increased intake of low-fat dairy products but decreased with an increased intake of high-fat dairy products [75]. While the exact cause of this relationship is unknown, the relationship between dairy and PD risk may be explained by its effect on serum urate levels (higher serum urate levels are associated with a lower risk of PD). As a result, Hughes et al. [75] hypothesized that the protein found in dairy products might decrease serum urate levels, while saturated fat may increase serum urate levels. The reliance on patient-reported PD diagnosis and disease severity indicators limits the generalizability of the study by Mischley et al. [73]. Additionally, the primary author’s ownership of the tool used by patients to report outcomes related to PD severity, the PRO-PD, increases the study’s risk of bias.

Additionally, examining specific food groups through factor analysis can describe dietary patterns linked to disease risk. Okubo et al. [77] utilized factor analysis to examine the dietary pattern of 249 PD cases and 368 controls recruited from eleven different hospitals in Japan. Okubo et al. [77] identified three different dietary patterns (healthy, light meal, and western) associated with PD risk. The healthy dietary pattern included increased intake of vegetables, seafood, and tea with a low intake of alcohol [77]. The Western diet was rich in beef, pork, chicken, vegetable oil, and salt. The light meal dietary pattern was rich in bread, noodles, dairy products, fruit, soft drinks, and sugar [77]. Overall, Okubo et al. [77] found that greater adherence to a healthy dietary pattern trended with a reduced risk of PD among participants. Nevertheless, the study methodology (the inclusion of non-matched controls and study questionnaires not validated for the study population) limits the generalizability of the results [77]. While both Mischley et al. [73] and Okubo et al. [77] examined specific food groups to characterize a dietary pattern associated with positive PD outcomes, the studies lack comparability to studies examining adherence to previously defined dietary patterns. Besides examining individual food groups, researchers examine healthy dietary patterns in terms of diet quality. Molsberry et al. [78] measured diet quality using the AHEI scoring system among 17,400 participants from the Nurses’ Health Study and the Health Professionals Follow-up Study. Participants with greater AHEI scores were less likely to develop symptoms of prodromal PD. In contrast to Molsberry et al. [78], Sääksjärvi et al. [79] found no relationship between AHEI scores and risk of PD in a cohort of 4524 Finnish men and women. Out of the 4524 participants, 85 PD cases were recorded [79]. Sääksjärvi et al. [79] used a modified version of the AHEI due to the absence of data on participant intake of multivitamin supplements and alcohol [79]. While the exclusion of supplement and alcohol intake could account for the difference in the two studies’ results, a large European cohort study by Peters et al. [80] further observed no relationship between lifetime alcohol consumption or the type of alcoholic beverage consumed and PD risk. Furthermore, the difference in the results of the two studies could be related to the different study populations and the definitions of PD utilized.

In addition to the AHEI, researchers can assess diet quality using the Dietary Screening Tool (DST)—a questionnaire designed to measure dietary intake and eating behavior [81]. In a cohort study, Liu et al. [81] utilized the DST to examine diet quality and PD risk among 3653 men and women over 65 years old residing in the US. After almost seven years of follow-up, 47 participants developed PD [81]. Overall, Liu et al. [81] observed a significant inverse relationship between better diet quality and PD risk. As a result, the study supports the benefits of adhering to a healthy dietary pattern to reduce the risk of PD. Nevertheless, more research is needed to explore the impact of diet quality on PD as measured by DST among different populations and geographic regions.

While the benefits of adhering to a generally healthy diet can be measured using diet quality indexes, researchers also explore the role of specific dietary patterns, such as the DASH diet, in PD. Agarwal et al. [82] conducted an observational study in a cohort of 706 US men and women. In the study, Agarwal et al. [82] found no relationship between adherence to the DASH diet and PD risk. Though this study suggests no association between the DASH diet and PD, the cohort primarily consisted of elderly females. Thus, more research is required to examine the DASH diet in larger diverse cohorts.

While the studies discussed so far relied primarily on observational data, Hegelmaier et al. [83] conducted a cross-sectional study and clinical trial. The study built upon previous research linking PD to gut microbiome dysbiosis [84]. First, Hegelmaier et al. [83] examined the gut microbiome of 54 participants with idiopathic PD and 32 healthy controls in Germany. In agreement with previous research, Hegelmaier et al. [83] found differences between the microbiome of participants with PD and healthy controls. Second, a subset of sixteen of the PD patients received an ovo-lacto-vegetarian diet (*n* = 16) for fourteen days [83]. Ten of the sixteen participants received the ovo-lacto-vegetarian diet combined with an enema intervention for eight days [83]. Although both the diet-only and combined intervention groups had significant improvements in motor symptoms (UPDRS Part III), the combined intervention group had the most significant score improvements at the end of the 14 days [83]. Compared to baseline, the combined intervention group’s mean levodopa dosage was lower one-year post-intervention, while the diet-only group’s mean dosage had increased [83]. While the study supports the role of diet in PD, the study’s small sample size and lack of diversity limit the generalizability of the data. More research needs to be conducted to solidify the benefits of diet on modifying the gut microbiome in PD and alleviating motor symptoms.

Overall, the articles mentioned to this point explored the relationship between following a general healthy dietary pattern and PD development and progression. Mischley et al. [73], Okubo et al. [77], Molsberry et al. [78], and Liu et al. [81] support the benefits of following a general healthy dietary pattern and reduced PD risk or severity. Furthermore, the clinical trial by Hegelmaier et al. [83] provides evidence that a healthy dietary pattern may modify PD severity. Nevertheless, the comparability and generalizability of the studies have several limitations, including the different definitions used to measure adherence to a healthy dietary pattern. The remainder of this review focuses on the evidence for four specific dietary patterns: the protein-restricted diet (PRD), the ketogenic diet (KD), the Mediterranean diet (MD), and the Mediterranean-DASH Diet Intervention for Neurodegenerative Delay (MIND).

## 3. Protein-Restricted Diet

While some dietary patterns focus on the distribution of different food groups in an individual’s diet, other dietary patterns focus on the distribution of macronutrients, such as protein. Previous research linked dietary protein to levodopa bioavailability based on both substances utilizing the same large neutral amino acid transporter for absorption in the small intestine and transport across the blood–brain barrier [85,86,87]. The purpose of a PRD is to improve the bioavailability of levodopa by limiting protein consumption [87,88]. Although previous studies examining the role of a PRD on PD indicated a beneficial impact on disease management, the studies’ small sample sizes limit the generalizability of the data [86,87,88,89]. 

### 3.1. Defining a Protein-Restricted Diet

Since a PRD aims to reduce complications from drug-nutrient interactions, two different definitions predominate scientific literature. First, a low-protein diet is a dietary pattern that limits daily protein intake to 0.8 g/kg of an individual’s body weight [87,89]. Second, a protein redistributed diet may or may not require participants to adhere to the maximum daily protein intake of 0.8 g/kg. Instead, the definition of a protein redistributed diet focuses on the timing of protein intake, limiting protein intake during the morning and afternoon to 7 g and allowing unlimited protein intake during the evening meal until bedtime [87].

While the daily protein limit of 0.8 g/kg in the low-protein diet is based on the Recommended Dietary Allowance (RDA) [89], some studies have raised concerns that 0.8 g/kg/day may not be sufficient to meet the protein needs of individuals with PD. For example, Silva et al. [90] observed a negative nitrogen balance among seventeen participants with PD consuming an average of 1.1 g/kg of protein per day. As a result, some studies have examined the impact of energy- or protein-rich nutrition supplements in PD patients adhering to a PRD.

### 3.2. The Impact of a Protein-Restricted Diet on PD

Over the years, several studies have examined the impact of a PRD on PD. In an Italian observational study, Barichella et al. [91] examined adherence to a protein redistributed diet on PD severity in 600 PD cases and 600 controls recruited from a single center. Compared to the age- and gender-matched control group, participants with PD had significantly lower BMI (26.2 kg/m2 vs. 28.5 kg/m2; *p* < 0.001), greater energy intake (31.3 kcal/kg vs. 26.7 kcal/kg; *p* < 0.001), and greater protein intake (1.2 g/kg vs. 1.0 g/kg; *p* < 0.001) [91]. Barichella et al. [91] observed that PD participants with greater adherence to a protein redistributed diet received lower levodopa doses and experienced fewer motor symptoms fluctuations. Additionally, Barichella et al. [91] found a direct association between increased protein intake by 10 g—over the RDA of 0.8 g/kg/day—was directly linked to an increase in levodopa dosage. As a result, the study’s data suggest that adherence to a protein redistributed diet may assist individuals with PD in managing motor fluctuations linked to drug-nutrient interactions. While the study did not raise any concerns about the nutritional safety of adhering to a PRD, the study center includes nutrition services as part of the disease management of patients receiving diet-related recommendations. 

Because restrictive diets may worsen the nutritional status of individuals with neurological disease, Cucca et al. [92] conducted a randomized control trial to examine the safety of an amino acid supplement in twenty-two men and women receiving levodopa therapy and following a protein redistributed diet. In the Italian study, participants were randomized to receive two doses of either an amino acid supplement (4 g of essential polar amino acids per dose) or a placebo twice a day for six months [92]. While neither group experienced a significant change in levodopa dosage, motor fluctuations, or UPDRS Part III score during the study, both groups experienced a significant improvement in nutrition status as measured by the Mini Nutrition Assessment [92]. As a result, the study indicated that individuals with PD could safely consume an amino acid supplement to prevent the adverse effects of a PRD on nutrition status. Nevertheless, the small sample size limits the generalizability of the study [92]. The strict timing of supplement and levodopa intake prevents the study results from informing researchers on the safety and effectiveness of supplement consumption at other times of the day. Furthermore, the strict timing contributed to the study’s high drop-out rate. Two out of the eight participants who withdrew from the study cited complications such as nausea and early satiety from the amino acid supplement as their reasoning for withdrawing from the study [92]. Therefore, more research is needed to confirm the benefits of amino acid supplements in counteracting the adverse effects of PRD in PD.

Because the drug-nutrient interaction between protein and levodopa is the basis behind a PRD, Virmani et al. [93] conducted an observational study to examine the impact of “protein interactions with levodopa (PIL)” on motor fluctuations in 1037 individuals with PD in the US. The researchers considered PIL to have occurred if participants reported motor fluctuations following the consumption of a meal containing protein-rich foods, such as dairy, eggs, and meat [93]. Virmani et al. [93] noted that 5.9% of levodopa therapy participants reported PIL. As a result, the study’s data indicates that a PRD may not be necessary for most individuals with PD on levodopa therapy. Virmani et al. [93] also observed that PIL developed on average 12.9 years after the initial appearance of motor symptoms and 7.9 years after levodopa therapy initiation. Therefore, the study’s data indicates that a PRD may not be necessitated during the initial stages of PD and levodopa therapy. Regardless, the small proportion (*n* = 52) of participants reporting PIL limits the generalizability of the study. The study also highlighted concerns about the safety of a PRD, with 12 out of 20 participants reporting weight loss following diet modification. Given the small number of participants reporting PIL and the potential for weight loss following a PRD, the results of Virmani et al. [93] highlight the need for more research on the benefits and risk of following a PRD among individuals with PD.

Overall, the studies discussed build upon previous research indicating that a PRD may be beneficial in managing motor fluctuations of individuals with PD on levodopa therapy. The study by Barichella et al. [91] supports the benefits of a PRD on motor fluctuations in PD and indicates that future research must explore the impact of regular nutrition services in preventing the diet’s adverse effects. Additionally, the data by Cucca et al. [92] indicate that polar amino acids supplements may serve as an effective strategy for maintaining nutrition status in an individual with PD while on a PRD. Lastly, the study by Virmani et al. [93] indicates that a PRD may not be necessary for all individuals with PD receiving levodopa therapy, especially during the initial stages of PD.

## 4. Ketogenic Diet

While a PRD diet focuses on the quantity and distribution of protein in an individual’s diet, other dietary patterns focus on the distribution of dietary carbohydrates and fats. The KD is a dietary pattern low in carbohydrates and rich in fat [94]. Since 1921, the KD has served as a potential treatment for epilepsy [94,95]. Recently, research has begun to explore the potential benefits of the KD on type 2 diabetes [96] and Alzheimer’s Disease [97,98,99,100].

### 4.1. Defining the Ketogenic Diet

According to the Academy of Nutrition and Dietetics [101], the term “ketogenic diet” refers to any dietary pattern expected to promote a ketogenic state in an individual. From a physiological perspective, a very-low-carbohydrate diet decreases the body’s supply of glucose and results in a metabolic shift from using glucose as its primary fuel source to using fatty acids [97]. As a result, the degree of ketogenesis—the breakdown of fatty acids into ketone bodies (acetoacetate, acetone, and β-hydroxybutyrate)—increases [98]. During this time, the body maintains blood glucose levels and synthesizes glucose from either amino acids or glycerol through a process known as gluconeogenesis [102]. As the level of ketone bodies in the bloodstream rises, the body enters a state of ketosis.

While the KD broadly encompasses any dietary pattern predicted to induce a ketogenic state [101], some clinicians and researchers utilize stricter definitions. The “classic” or ‘traditional’ KD consists of a ratio of 4:1 or 3:1 (dietary fat: dietary protein and carbohydrates) [101]. The modified Atkins diet is less restrictive than the classic KD and utilizes a ratio of 1:1 [103]. Other versions of a KD include the medium-chain triglyceride KD and low glycemic index treatment, allowing the user to consume slightly more carbohydrates than the classic KD [104].

Additionally, the KD’s restrictive nature necessitates the involvement of a patient’s Physician and Registered Dietitian Nutritionist (RDN), particularly when a KD is prescribed for the treatment of epilepsy [101,104]. Furthermore, compliance with a KD may be hindered by its associated short- and long-term side effects such as anemia, constipation, cardiomyopathy, decreased appetite, hepatitis, nausea, vomiting, nephrolithiasis, and pancreatitis [94,98].

### 4.2. The Impact of the Ketogenic Diet on PD

Several studies examined the potential impact of the KD on PD. In an 8-week clinical trial conducted in New Zealand, Philips et al. [105] randomized participants to follow either a low-fat diet (*n* = 23) or a KD (*n* = 24) [105]. Both diet plans provided participants with the same amount of protein (1.0 g/kg/day) and included a weekly shopping list, daily menu sets, and recipes [105]. While both intervention groups experienced significant improvements in MDS-UPDRS Part I, Part II, and Part III scores, only the KD group experienced a significant improvement in MDS-UPDRS Part IV scores [105]. As a result, the study by Philips et al. [105] indicates that short-term use of either a low-fat diet or KD could alleviate PD symptoms. Because data was not collected on the effect of either diet post-intervention, more research is needed to examine the long-term implications of a low-fat diet or KD after diet cessation in PD. The occurrence of weight loss among participants in both diet groups and exacerbated tremor or rigidity among patients in the KD group also highlights the need for more research on the safety of long-term adherence to a KD in individuals with PD [105].

In an 8-week clinical trial in the US, Krikorian et al. [106] examined the impact of the KD among eighteen participants with PD-mild cognitive impairment (MCI). Participants were randomized to follow a low-carbohydrate (ketogenic) diet (*n* = 10) or a high-carbohydrate diet (*n* = 8) [106]. While participants following the KD had significant improvements in cognitive performance compared to the high-carbohydrate group, no difference emerged between the groups in MDS-UPDRS Part III after the intervention [106]. Furthermore, the KD group experienced a significant reduction in body weight, and Krikorian et al. [106] noted a relationship between weight loss and improvements in cognitive function. Therefore, more research is needed to explore the safety of long-term adherence to the KD and its relationship with weight loss and cognition.

Both Philips et al. [105] and Krikorian et al. [106] examined the potential role of short-term adherence to a KD among individuals with PD. While both studies indicate that a low-carbohydrate (ketogenic) diet may alleviate some PD symptoms, the researchers found conflicting results on the impact of a KD on motor symptoms (MDS-UPDRS Part III). Therefore, more research is needed to examine this relationship. Furthermore, Philips et al. [105] and Krikorian et al. [106] reported weight loss among participants following the KD. Because previous studies have linked weight loss to malnutrition risk and adverse outcomes for individuals with PD [38], more research is needed to explore the impact of a ketogenic dietary intervention on the nutrition status of individuals with PD.

In a longer (3-month) clinical trial in Turkey, Koyuncu et al. [107] examined the effect of the KD on voice quality among 74 men and women with PD who were not receiving medication for PD treatment. Participants were randomized to follow either a regular diet (*n* = 37) or a KD (*n* = 37) [107]. Unlike the regular diet group, participants in the KD group had significant improvements in voice quality as measured by the Voice Handicap Index-10, a scoring system that uses self-reported measures of voice quality [107]. While the study indicates that a KD may improve voice quality in PD, the lack of information on participant KD training and the degree of participant adherence limits the generalizability of the study [107].

While the studies discussed indicate that adherence to a KD may improve various PD symptoms, the studies’ small sample sizes and short intervention periods limit their generalizability. Furthermore, Philips et al. [105] and Krikorian et al. [106] reported weight loss among participants with PD following a KD. Because previous research indicates that PD patients are at greater risk of developing malnutrition, more research is needed to explore the long-term implications of adherence to a KD on the nutritional status and quality of life of PD patients. Additionally, Philips et al. [105] and Krikorian et al. [106] utilized nutrition education and counseling to prepare participants and their caregivers for implementing a KD. In light of the role of an RDN in KD therapy in epilepsy, future research should also examine the role of RDNs in improving KD compliance and safety among individuals with PD.

## 5. Mediterranean Diet

The MD may play a beneficial role in disease prevention, and several studies support its role in reducing all-cause mortality [108,109] along with the risk of cancer [110], diabetes [111], stroke [112], and cardiovascular disease [112]. Moreover, the MD may positively influence health-related biomarkers such as high-density lipoproteins [112,113], triglycerides [112,114], blood pressure [112], waist circumference [112], and insulin resistance [115]. Recently, scientific evidence is emerging to support the beneficial role of the MD on depression [116,117,118], Alzheimer’s disease [62], and neurodegeneration.

### 5.1. Defining the Mediterranean Diet

First identified by Ancel Keys, the definition of the MD has changed over the years [119]. As a result, the definition of the MD varies from study to study—making it difficult to compare results and draw conclusions [120,121]. Broadly, the MD represents a dietary pattern rich in fruits, vegetables, legumes, cereals, nuts, fish, and monounsaturated fatty acids with moderate alcohol intake and low intake of dairy products and red meats [122].

Previously, several studies explored the definition of the MD to clarify its meaning. Davis et al. [122] noted differences across research studies in the number of serving sizes and the number of grams for different components of the MD. Furthermore, Abdelhamid et al. [120] conducted a systematic review of seventy-four primary research articles and observed considerable variability in the methods for defining food group categories and calculating the MD score. The observed variation likely stemmed from the MD score’s reliance on specific population and study food group category means over absolute setpoints [120]. Zaragoza-Martí et al. [123] evaluated the quality of twenty-eight different MD scores based on the Scientific Advisory Committee of the Medical Outcomes Trust’s criteria [123]. The authors noted that several studies considered the method developed by Trichopoulou et al. [124] in 1995 to be the gold standard because it was developed first; however, evidence was insufficient for all MD scores with the methods developed by Panagiotakos et al. [125], Buckland et al. [126], and Sotos-Prieto et al. [127] possessing the most evidence [123].

Similar to previous studies, variations exist in how studies calculated MD adherence in this review. Five of the studies [78,128,129,130,131] used the MD score created by Trichopoulou et al. [132] or a variation of that method, while three studies [82,130,133] used the MD score created by Parganiotakos et al. [125] or a variant of that method. Trichopoulou et al. [132] MD score builds upon the score created by Trichopoulou et al. [124] but updated the previous score to include dietary fish intake. The score rewards participants for consuming more significant amounts of cereal, fish, fruit, legumes, nuts, and vegetables and lower amounts of dairy products, meat, and poultry [132]. Trichopoulou et al. [132] observed a positive association between higher MD score and all-cause mortality, coronary heart disease mortality, and cancer mortality. Furthermore, the results maintained their significance after controlling for age, sex, education, smoking status, BMI, waist-to-hip ratio, energy expenditure, energy intake, egg consumption, and potato consumption [132].

### 5.2. Impact of the Mediterranean Diet on PD

Currently, studies examining the role of the MD on PD have yielded mixed results. In a study of 41,715 middle-aged Swedish women, Yin et al. [131] observed a significant inverse relationship between MD adherence and risk of PD and noted an 11% reduction in PD risk per unit increase in MD score. Agarwal et al. [82] observed a 3% reduction in PD risk per unit increase in MD score. Additionally, greater adherence to the MD was associated with slower PD progression as measured by the UPDRS [82]. The differences in per unit risk reduction may be accounted for by differences in participant gender, geographical location, and mean follow-up period [82]. Compared to the incidence of 101 PD cases in Yin et al. [131], Agarwal et al. [82] documented 302 PD cases. The difference in PD incidence may be explained by Agarwal et al. [82] using trained clinicians to diagnose PD and Yin et al. [131] utilizing hospital records to confirm PD diagnosis. In contrast to Yin et al. [131] and Agarwal et al. [82], Maraki et al. [133] found no relationship between MD adherence and PD risk among the 1765 participants (34 PD cases) enrolled in the Hellenic Longitudinal Investigation of Aging and Diet in Greece. Nevertheless, Maraki et al. [133] noted that their analysis might be underpowered by the documentation of only thirty-four PD cases.

When examining the probability of prodromal PD instead of clinical PD, Maraki et al. [133] found an inverse relationship between MD score and the probability of prodromal PD. Similarly, Molsberry et al. [78] observed a reduced risk of prodromal PD with greater MD adherence. Studies examining the role of the MD on the age of onset of PD have yielded mixed results. In a US case–control study of 257 cases and 198 controls, Alcalay et al. [128] observed that greater adherence to the MD was associated with later onset of PD. Conversely, Cassani et al. [129] found no relationship between MD adherence and age at onset of PD when examining 600 cases and 600 controls in Italy [129]. While both studies calculated MD scores using the method established by Trichopoulou et al. [132], they occurred in different geographical regions, and local food availability and food preferences could have confounded results [128,129]. Additionally, Cassani et al. [129] observed significantly greater potato consumption among the PD group, but it is unclear how potato consumption affected MD score calculation. For instance, if potato consumption were factored into the vegetable intake, PD patients would have received higher MD scores for increased potato consumption despite Trichopoulou et al. [132] excluding potato intake from the MD score and associating it with a greater risk of all-cause mortality. 

Metcalfe-Roach et al. [130] examined the role of the MD on the age of onset of PD among Canadian men and women using the original MD score developed by Trichopoulou et al. [132] and the “Greek” MD score developed by Panagiotakos et al. [134]. Compared to the “original” MD score, greater adherence to the “Greek” MD score was more strongly associated with later age at onset among 167 PD cases and 119 controls [130]. Interestingly, the MD score developed by Panagiotakos et al. [134] rewards participants for the consumption of potatoes, limiting the comparability of studies using the different scoring methods. Furthermore, the exact impact of potato consumption on health outcomes is unclear [135]. Overall, the results of Metcalfe-Roach et al. [130] highlight the importance of using consistent definitions of the MD to increase the comparability of study results.

Furthermore, two articles examined the MD in a single-center, 10-week randomized control trial (RCT) of eighty idiopathic PD patients in Iran [136,137]. The first article examined cognitive function and randomized participants to follow the MD (*n* = 40) or healthy dietary recommendations (*n* = 40) [136]. At the end of the study, 35 participants remained in each group [136]. Compared to the control group, participants following the MD had significantly improved scores for executive function, language, attention, concentration, and active memory [136]. The second article examined disease severity using the MDS-UPDRS and randomized participants to follow either an MD (*n* = 40) or the traditional Iranian diet (*n* = 40) [137]. At the end of the study, 36 participants remained in the intervention group, and 34 participants remained in the control group [137]. Compared to the control group, the MD group experienced greater serum total antioxidant capacity and decreased disease severity [137]. While both studies support the benefits of the MD in PD patients, the inclusion of PD patients from a single center limits the studies’ generalizability to other parts of Iran and the world.

In summary, the seven case–control and cohort studies [78,82,128,129,130,131,133] yielded mixed results on the benefits of the MD in PD prevention and progression. The MD was associated with a reduced risk of clinical PD in two studies [82,131] (compared to one [133]), reduced risk of prodromal PD in two studies [78,133], later age of onset in two studies [128,130] (compared to one [129]). Differences in mean population dietary patterns and methods of calculating MD scores could account for these discrepancies. Additionally, the recruitment of participants from a single center limits the positive results obtained in the two RCT articles’ [136,137]. Therefore, more research is needed to examine the benefits of the MD in PD.

## 6. MIND Diet

Developed to protect against neurodegeneration, the MIND diet combines the MD and DASH dietary patterns into a single dietary pattern [138]. Previous research indicates that greater adherence to the MIND diet is associated with a reduced risk of all-cause mortality [139], psychological disorders [140,141], and cognitive decline [142,143]. Additionally, the MIND diet may play a beneficial role in reducing the risk of another neurodegenerative disease, Alzheimer’s Disease [144]. While the protective role of the MIND diet in cognitive decline and Alzheimer’s Disease makes it of interest in PD, the MIND diet’s potential impact on PD symptoms and an individual’s physical function increases its relevance to PD. For instance, a recent study by Talegawkar et al. [145] observed that greater adherence to the MIND diet was associated with better physical function and grip strength in US adults over 60 years old. This portion of the review will examine the impact of the MIND diet on PD.

### 6.1. Defining the MIND Diet

Morris et al. [138] developed the MIND diet and its scoring method to reward participants for consuming foods associated explicitly with neuroprotection. As a result, the MIND diet retains the focus of the MD and DASH on plant-based foods while additionally emphasizing the consumption of green leafy vegetables and berries [138].

### 6.2. Impact of the MIND Diet on PD

Both studies [82,130] examining the MIND diet observed a decreased risk of developing PD. Agarwal et al. [82] observed a 13% reduction in PD risk among men and women with greater adherence to the MIND diet. Likewise, Metcalfe-Roach et al. [130] observed an association between greater adherence to the MIND diet and later age at onset of PD; however, the relationship was most substantial in the female subgroup. The more substantial reduction in PD risk among females could be related to the “berry” component of the MIND diet score. Sääksjärvi et al. [79] observed that the increased intake of berries was associated with an increased risk of PD in men but a decreased risk in women. Furthermore, both Agarwal et al. [82] and Metcalfe-Roach et al. [130] observed a greater adherence to the MIND diet to be more protective against PD than the MD. Overall, both the work by Agarwal et al. [82] and Metcalfe-Roach et al. [130] highlight the benefits of greater adherence to the MIND diet in PD prevention; however, more research is needed to confirm the relationship.

## 7. Summary

**Figure 1:**
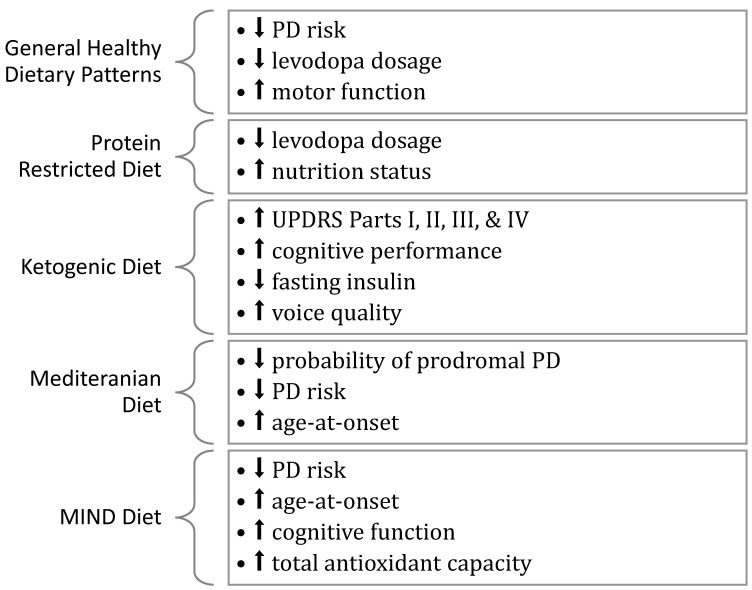
Potential benefits of different dietary patterns for individuals with PD. Upward arrows (⬆) indicate the diet increased the following variable, while downward arrows (⬇) indicate that the diet decreased the following variable.

In summary, this review examined the role of diet and dietary patterns in PD in five different categories: general, PRD, KD, MD, and MIND. Table 1 provides a summary of the results of the observational studies and Table 2 provides a summary of the clinical trials included in this review. The potential benefits of different dietary patterns for individuals with PD are highlighted in Figure 1. Four observational studies [73,77,78,81] and one clinical trial [83] support the benefits of following a general healthy dietary pattern in reducing PD risk or severity. While the three PRD studies [91,92,93] support the beneficial role of a PRD in managing motor fluctuations of individuals with PD on levodopa therapy, more research is needed to clarify when a PRD is appropriate for alleviating motor fluctuations among individuals with PD. Similarly, the three KD studies [105,106,107] indicate that adherence to a KD may improve various PD symptoms. Nevertheless, the small sample sizes, short periods, and diet’s adverse effects require more research on the role of PRD and KD in PD. Eight articles [78,82,128,129,130,131,133,136,137] indicate that the MD may prevent PD and progression. Lastly, both MIND diet studies [82,130] support the role of the MIND diet in PD prevention. While most of the included studies support the role of diet and dietary patterns in reducing PD risk, progression, or severity, inconsistent results and small sample sizes highlight the need for further research before making nutritional recommendations. Therefore, more research is needed to explore the impact of specific dietary patterns on PD along and their potential benefits and risks.

## 8. Conclusions

Overall, PD is a neurodegenerative disorder associated with diminished nutrition status and quality of life. Since no preventative or curative therapy for PD exists currently, nutrition and diet represent modifiable risk factors for reducing disease risk. As the single-nutrient approach yields inconsistent results on the role of diet in PD, this review analyzed studies investigating the role of diet from a holistic approach. Although research on a general healthy dietary pattern, a PRD, a KD, an MD, and a MIND diet yielded mixed results, most studies examined in this paper support the role of diet and dietary patterns in reducing the risk of PD or alleviating PD severity. Nevertheless, more research is needed to examine the relationship and explore the impact of specific dietary patterns.

## Figures and Tables

**Table 1:** Summary of Observational Study Results on the Impact of Diet and Dietary Patterns on PD.

Citation	Year Published	Study Type	Diet Type	*n*	Location	Key Results
Alcalay et al. [128]	2012	Case-Control	MD	257 cases 198 controls	United States	Greater adherence to the MD was associated with a reduced risk of PD and later age-at-onset of PD.
Okubo et al. [77]	2012	Case-Control	Healthy Western Light Meal	249 cases 368 controls	Japan	The healthy dietary pattern was associated with a reduced risk of PD, but not statistically significant (*p* = 0.06). The light meal and Western dietary patterns were not associated with PD risk.
Sääksjärvi et al. [79]	2012	Cohort	AHEI	4524 (85 cases)	Finland	Adherence to the AHEI was not associated with PD risk. Greater intake of berries was associated with a reduced risk of PD in women but was associated with an increased risk among men.
Virmani et al. [93]	2016	Cohort	PRD	1037 (1037 cases)	United States	Only 5.9% of participants on levodopa reported PIL. Only 20 participants reported following a PRD.
Barichella et al. [91]	2017	Case-Control	PRD	600 cases 600 controls	Italy	Adherence to a PRD was associated with a lower levodopa dosage. Protein intake was not associated with levodopa-related motor complications. An intake of 10 g protein over 0.8 g/kg/day was associated with an increase in levodopa dosage by 0.7 mg/kg.
Cassani et al. [129]	2017	Case-Control	MD	600 cases 600 controls	Italy	No difference in adherence to the MD existed between cases and controls. Adherence to the MD was not associated with disease duration, or age-at-onset.
Mischley et al. [73]	2017	Cross-Sectional	General	1053 (1053 cases)	United States	A plant/fish based dietary pattern was associated with a reduced rate of PD progression. Foods associated with a reduced rate: fresh vegetables, fresh fruit, nuts, seeds, fish, olive oil, coconut oil, and wine. Foods associated with an increased rate: canned vegetables, canned fruit, beef, fried food, cheese, yogurt, ice cream, and soda.
Agarwal et al. [82]	2018	Cohort	DASH MD MIND	706 (302 cases)	United States	The DASH Diet was not associated with PD risk. Both the MIND diet and the MD were associated with a reduced risk of PD, with the MIND diet having the strongest relationship to PD risk. Each unit increase in the MIND diet score was associated with a 13% reduction in PD risk.
Maraki et al. [133]	2018	Cohort	MD	1765 (34 cases)	Greece	Adherence to the MD was associated with a lower probability of prodromal PD. The study’s results remained unchanged after excluding constipation as a feature of prodromal PD.
Liu et al. [81]	2020	Cohort	DST	3653 (47 cases)	United States	Greater diet quality was associated with a significantly reduced risk of PD.
Molsberry et al. [78]	2020	Cohort	MD AHEI	17,400	United States	Greater adherence to both the MD and AHEI was associated with a reduced risk of developing features of prodromal PD.
Metcalfe-Roach et al. [130]	2021	Case-Control	MD MIND	167 cases 119 controls	Canada	Greater adherence to the MIND diet or the Greek MD was associated with later age of onset of PD. The relationship was stronger for the MIND diet than the MD. The relationship between the MIND diet and age of onset was strongest among women, while the relationship between the MD (Panagiotakos et al. [134]) was strongest among men.
Yin et al. [131]	2021	Cohort	MD	41,715 (101 cases)	Sweden	Greater adherence to the MD was associated with a reduced risk of PD. Each unit increase in MD score was associated with an 11% reduction in PD risk.

Note: Table abbreviations include Dietary Approaches to Stop Hypertension (DASH); Dietary Screening Tool (DST); Mediterranean-DASH Intervention for Neurodegenerative Delay (MIND); Mediterranean Diet (MD); Parkinson’s Disease (PD); Protein Interactions with Levodopa (PIL); Protein Restricted Diet (PRD); Sample Size (*n*).

**Table 2:** Summary of Clinical Study Results on the Impact of Diet and Dietary Patterns on PD.

Citation	Year Published	Diet Manipulation	Length	Location	Key Results
General Dietary Patterns					
Hegelmaier et al. [83]	2020	Individuals with PD were randomized to receive either an enema for 8 days and an ovo-lacto-vegetarian diet (*n* = 10) or diet only (*n* = 6).	2 weeks	Germany	Compared to baseline: Compared to the diet only group, the combined treatment group had significant improvements in UPDRS III and daily levodopa dose a year after the intervention.
PRD					
Cucca et al. [92]	2015	Individuals with PD on a PRD were randomized to consume either 16 g amino acid supplement (*n* = 12) or a placebo (*n* = 10) daily.	6 months	Italy	Compared to baseline: Neither group experienced a significant change in body weight, handgrip strength, levodopa dosage, or motor performance (UPDRS III) Both groups experienced a significant improvement in nutrition status Both groups experienced a decrease in insulin sensitivity, with the greatest decrease occurring in the placebo group
KD					
Phillips et al. [105]	2018	Individuals with PD were randomized to follow either a low-fat diet (*n* = 23) or a KD (*n* = 24).	8 weeks	New Zealand	Compared to baseline: Both the KD and low-fat groups experienced a significant improvement in UPDRS Part I, II, and III. Furthermore, the KD group experienced a greater improvement in UPDRS Part I than the low-fat group. Only the KD group experienced a significant improvement in UPDRS Part IV.
Krikorian et al. [106]	2019	Individuals with PD randomized to follow either a low carbohydrate (*n* = 10) or a high carbohydrate (*n* = 8) diet.	8 weeks	United States	Compared to baseline: The low carbohydrate group experienced a significant reduction in body weight, waist circumference, and fasting insulin along with an increase in β-hydroxybutyrate. The high carbohydrate group also experienced a decrease in fasting insulin. Compared to the high carbohydrate group, the low carbohydrate group experienced a significant improvement in cognitive performance. Neither group experienced a change in motor function (UPDRS Part III).
Koyuncu et al. [107]	2020	Individuals with PD were randomized to follow either a KD (*n* = 37) or their regular diet (*n* = 37).	3 months	Turkey	Compared to baseline: The KD group experienced a significant improvement voice quality as measured by all ten components of the Voice Handicap Index-10. The control group experienced no change in voice quality.
MD					
Paknabad et al. [137]	2020	Individuals with PD were randomized to follow either a MD (*n* = 40) or the traditional Iranian diet (*n* = 40).	10 weeks	Iran	Compared to baseline: The MD group experienced an increase in total antioxidant capacity, while the control group experienced no significant change. The MD group also experienced a significant improvement in mentation, behavior, and mood; activities of daily living; and complications of therapy as measured by the MDS-UPDRS. This improvement remained significant when compared to the control group.
Paknahad et al. [136]	2020	Individuals with PD were randomized to follow either a MD (*n* = 40) or their regular diet (*n* = 40).	10 weeks	Iran	Compared to baseline: The MD group significantly increased their intake of protein and eicosapentanoic acid, while their intake of total energy, carbohydrates, and saturated fatty acids decreased The MD group also experienced improvements in components of the Montreal Cognitive Assessment (aspects of executive function; language score; attention, concentration, and working memory; total cognitive score)

Note: Table abbreviations include International Parkinson and Movement Disorder Society (MDS); Ketogenic Diet (KD); Sample Size (*n*); Unified Parkinson’s Disease Rating Scale (UPDRS).

## Data Availability

Not applicable.
